# Supplementary material for: A Humanized Yeast Phenomic Model of Deoxycytidine Kinase to Predict Genetic Buffering of Nucleoside Analog Cytotoxicity
Source: Genes (Basel). 2019 Sep 30;10(10):770. doi: 10.3390/genes10100770 (PMC6826991; doi:10.3390/genes10100770)
Supplement: Supplementary file 1 [file genes-10-00770-s001.zip › Additional_Files/Additional_File7_GOTermHeatmaps.zip!/Additional_File7_GOTermHeatmaps/telomeric_DNA_binding.pdf]

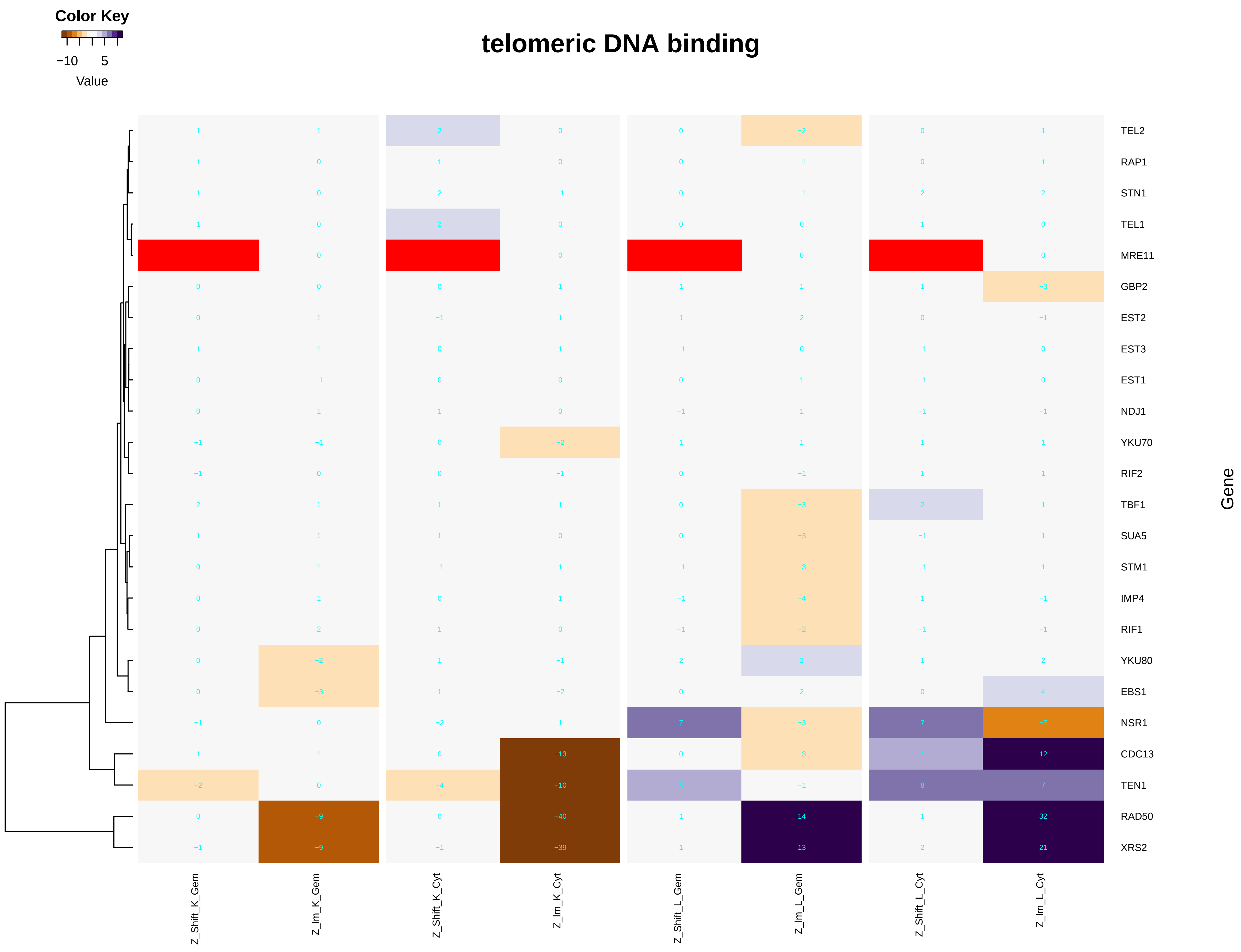
telomeric DNA binding
Color Key
−10
5
Value
TEL2
RAP1
STN1
TEL1
MRE11
GBP2
EST2
EST3
EST1
NDJ1
YKU70
RIF2
TBF1
SUA5
STM1
IMP4
RIF1
YKU80
EBS1
NSR1
CDC13
TEN1
RAD50
XRS2
Gene
Z_Shift_K_Gem
Z_Im_K_Gem
Z_Shift_K_Cyt
Z_Im_K_Cyt
Z_Shift_L_Gem
Z_Im_L_Gem
Z_Shift_L_Cyt
Z_Im_L_Cyt

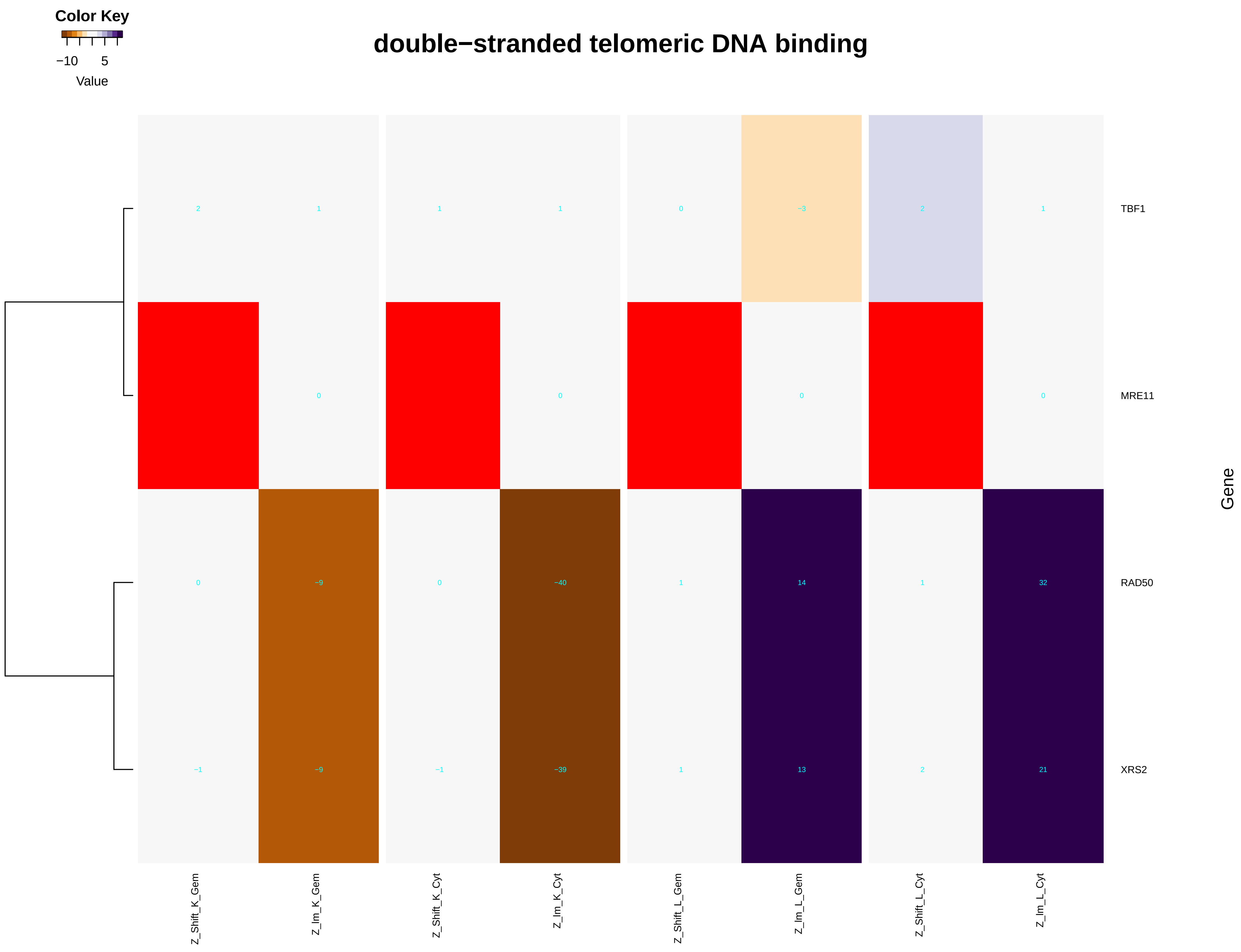

Color Key
−10
5
Value
double−stranded telomeric DNA binding
TBF1
MRE11
RAD50
XRS2
Gene
2
1
1
1
0
−3
2
1
0
0
0
0
0
−9
0
−40
1
14
1
32
−1
−9
−1
−39
1
13
2
21
Z_Shift_K_Gem
Z_Im_K_Gem
Z_Shift_K_Cyt
Z_Im_K_Cyt
Z_Shift_L_Gem
Z_Im_L_Gem
Z_Shift_L_Cyt
Z_Im_L_Cyt

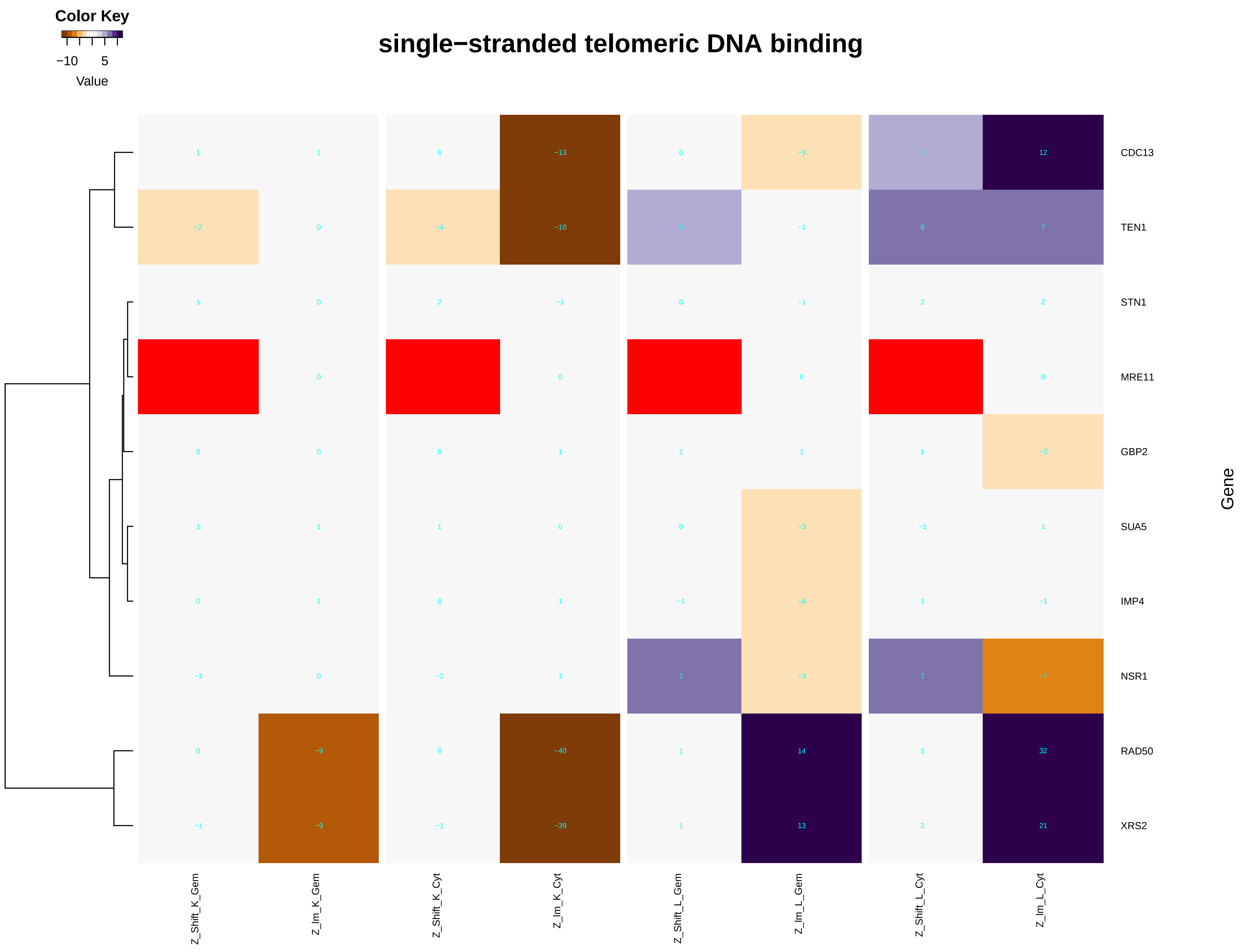
single−stranded telomeric DNA binding
Color Key
−10
5
Value
CDC13
TEN1
STN1
MRE11
GBP2
SUA5
IMP4
NSR1
RAD50
XRS2
Gene
Z_Shift_K_Gem
Z_Im_K_Gem
Z_Shift_K_Cyt
Z_Im_K_Cyt
Z_Shift_L_Gem
Z_Im_L_Gem
Z_Shift_L_Cyt
Z_Im_L_Cyt
